# Bringing Health and Fitness Data Together for Connected Health Care: Mobile Apps as Enablers of Interoperability

**DOI:** 10.2196/jmir.5094

**Published:** 2015-11-18

**Authors:** Valerie Gay, Peter Leijdekkers

**Affiliations:** ^1^ Faculty of Engineering and Information Technology University of Technology Sydney Broadway NSW Australia

**Keywords:** health informatics, connected health, pervasive and mobile computing, ubiquitous and mobile devices

## Abstract

**Background:**

A transformation is underway regarding how we deal with our health. Mobile devices make it possible to have continuous access to personal health information. Wearable devices, such as Fitbit and Apple’s smartwatch, can collect data continuously and provide insights into our health and fitness. However, lack of interoperability and the presence of data silos prevent users and health professionals from getting an integrated view of health and fitness data. To provide better health outcomes, a complete picture is needed which combines informal health and fitness data collected by the user together with official health records collected by health professionals. Mobile apps are well positioned to play an important role in the aggregation since they can tap into these official and informal health and data silos.

**Objective:**

The objective of this paper is to demonstrate that a mobile app can be used to aggregate health and fitness data and can enable interoperability. It discusses various technical interoperability challenges encountered while integrating data into one place.

**Methods:**

For 8 years, we have worked with third-party partners, including wearable device manufacturers, electronic health record providers, and app developers, to connect an Android app to their (wearable) devices, back-end servers, and systems.

**Results:**

The result of this research is a health and fitness app called myFitnessCompanion, which enables users to aggregate their data in one place. Over 6000 users use the app worldwide to aggregate their health and fitness data. It demonstrates that mobile apps can be used to enable interoperability. Challenges encountered in the research process included the different wireless protocols and standards used to communicate with wireless devices, the diversity of security and authorization protocols used to be able to exchange data with servers, and lack of standards usage, such as Health Level Seven, for medical information exchange.

**Conclusions:**

By limiting the negative effects of health data silos, mobile apps can offer a better holistic view of health and fitness data. Data can then be analyzed to offer better and more personalized advice and care.

## Introduction

Wearable health trackers such as the Jawbone UP [1] and Fitbit [2] have invaded the consumer market and make collection of personal health and fitness data ubiquitous. With the upcoming smartwatches supporting many features of the health trackers, these devices are becoming part of normal life and are integrated into a person’s daily routine. Improvements to wearable devices are occurring at a fast pace and newer models integrate improved sensors. For example, the Microsoft Band [3] includes a heart rate monitor, 3-axis accelerometer, gyro, ambient light sensor, skin temperature sensor, ultraviolet sensor, and galvanic skin response. Wearable devices come at a time when chronic diseases are on the rise, and at the same time governments are struggling with their health care budgets. Being able to collect biometric data in real time for a prolonged period make wearable devices a great tool to manage, or even prevent, some chronic diseases [4].

Wearable devices and mobile phone health apps can and will change health care by empowering users and educating them to take control of their health. Users are embracing them; according to the Intercontinental Marketing Services (IMS) Institute for Healthcare Informatics [5], as of 2015, there were 165,000 health-related mobile phone apps on Android and iPhone operating systems (iOS) and around 110,000 of these are for health and fitness. The IMS Health Institute [6] forecasts that the sales of wearable technology will grow to almost US $30 billion by 2018. According to Campbell [7], the health monitoring device industry is projected to exceed US $5 billion in 2016, largely due to the focus on patient engagement and prevention. The shift in users’ attitudes could lead to fewer doctor visits and the need for fewer tests. It also has the potential to give health professionals better insight into patients’ overall health and fitness.

There is an increasing amount of health- and fitness-related information that has been collected and stored in the cloud. However, the data usually reside in silos and in most cases health and fitness data are separated. For example, Fitbit stores all data generated by their trackers on their Fitbit server; the same applies to Jawbone, Withings [8], and iHealth [9]. Newcomers such as Google Fit [10] or Apple HealthKit [11] position themselves as integrators. However, will data stored in Apple HealthKit be available to Google Fit and vice versa? According to Mandi et al [12], these data streams will initially remain confined to their respective platforms and will have very limited ways to integrate with electronic health records (EHRs). To make it even more complicated, what about data stored in EHR systems that are controlled by governments?

Currently, there is no real integration of fitness-related data and health records stored in EHR systems. To provide better health outcomes and better patient engagement, a complete picture is needed which combines informal health and fitness data collected by the user, together with official health records collected by health professionals. By combining these two streams, the data can be analyzed using data analytics and health professional expertise to offer better personalized advice and care. There is good evidence that the integration can improve therapeutic management [13,14].

The objective of this paper is to demonstrate that a mobile app can be used to aggregate health and fitness data and can enable interoperability. It discusses various technical interoperability challenges encountered while integrating health and fitness data into one place. By limiting the negative effects of health data silos, mobile apps can offer a better holistic view of users' health and fitness data and give them more control over their data.

## Methods

Since 2007, we have worked with third-party companies to connect our Android app called myFitnessCompanion [15] to their sensors, wearable devices, EHR systems, and servers to collect and exchange health and fitness data. Initially, myFitnessCompanion only collected data coming directly from wireless sensors connected to the phone or by manual entry; the data were stored locally on the phone. Based on user feedback and comments, it became evident that our users wanted to control their data and aggregate their health and fitness data from other sources. Users also wanted to have the option to store all their data on one server (eg, Microsoft HealthVault [16]) or only keep it on their mobile device for privacy reasons. Observing this, we decided to develop our app into a health and fitness aggregator app. Today, myFitnessCompanion interacts with a wide range of wireless devices and wearable health trackers, and also aggregates data from third-party apps. It connects with Microsoft HealthVault, Google Fit, Fitbit, Withings, Jawbone, and iHealth servers as well as other EHR systems.

myFitnessCompanion was developed for Android devices and offers personalized exercise tracking and monitoring of biometric data, such as heart rate, respiration, body temperature, weight, food intake, blood pressure, cholesterol, asthma, blood glucose, and many more. It supports 15 different languages and has been commercially available on Google Play since 2011. Prior to the Android app, we developed a similar app using the Microsoft Windows Mobile 6.x platform. At that time, Microsoft did not offer an outlet like Google Play to distribute the app easily and, more disruptively, Microsoft discontinued support for Windows Mobile 6.x devices in 2011, which forced us to choose a new platform. We selected Android over Apple iOS, partially due to our experience with JAVA/C#, but more importantly because of the excellent Bluetooth support in the Android platform compared to iOS at that time.

**Figure 1:**
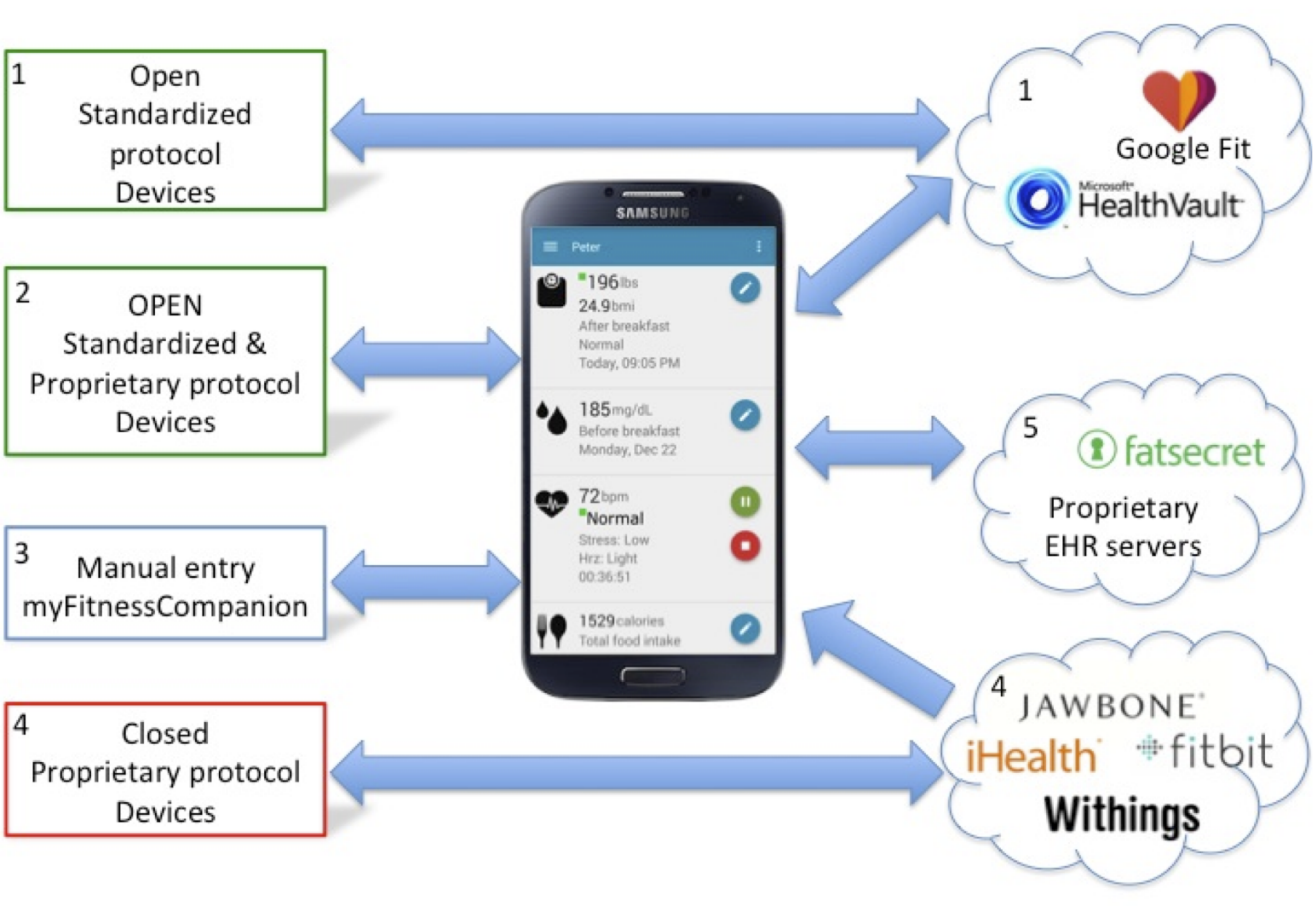
myFitnessCompanion ecosystem.

Our approach was to integrate off-the-shelf, commercially available devices. Simultaneously, we connected myFitnessCompanion with EHR servers, such as Microsoft HealthVault and Google Health (discontinued). These were the first EHR servers available to the general public. A major challenge was to keep up with the different Android operating system (OS) versions coming onto the market at a 3- to 6-month interval, resulting in a continuous process of updating the software to keep up to date with new Android devices and features. Figure 1 shows the ecosystem of myFitnessCompanion.

Devices supporting open standard protocols (Figure 1, box 1) are devices such as the Google Android Wear [17] smartwatches and fitness trackers that allow third-party developers to retrieve the data directly from the device. Fitness trackers such as the Mio LINK [18] or Garmin's Advanced and Adaptive Network Technology (ANT)+ Footpod [19] are open in the sense that they use standard open protocols to transfer health data using Bluetooth or ANT+. These devices are not necessarily connected to myFitnessCompanion and they upload their data directly to a server like Google Fit, which can then be retrieved by myFitnessCompanion. Users can also manually enter health data into Microsoft HealthVault or Google Fit, which is then automatically transferred to myFitnessCompanion.

Devices paired with myFitnessCompanion (Figure 1, box 2) refer to wireless Bluetooth or ANT+ sensors that are paired with myFitnessCompanion and whose data are directly streamed to the app. These include Bluetooth Smart heart rate monitors and blood pressure monitors. The devices implement an open standard. For example, Bluetooth Smart heart rate monitors from different vendors (eg, Zephyr HxM [20], Polar H7 [21], or Wahoo Blue [22]) all work seamlessly with the app without making adaptations for a specific vendor. Unfortunately, the majority of wireless devices implement a vendor-specific protocol. Sometimes the vendor makes the protocol available, which allows integration with myFitnessCompanion. Examples are A&D [23] blood pressure monitors and weight scales or FORA [24] blood glucose monitors. The disadvantage is that each device-specific software needs to be written to communicate and interpret the data transmitted by these devices.

Closed and proprietary wireless devices (Figure 1, box 4) do not allow third-party developers to communicate directly with the device. Although those devices use standard Bluetooth to communicate with a mobile device, the actual protocol and data format are not public. This makes it near impossible for third-party developers to integrate the device into their mobile app. Fitbit, Jawbone, Withings, and many other vendors follow this strategy and only allow third-party developers to obtain the data via their server through a public application programming interface (API). This means that these companies obtain all health and fitness data generated by their respective devices. It allows them to analyze and perform data mining, as well as sell the data to interested parties. Users have no choice but to hand over their health, fitness, and other personal data without knowing what is being done with it.

Websites such as MyFitnessPal [25] and FatSecret [26] collect health data by allowing users to input data directly or via their mobile app (Figure 1, box 5). These sites then allow third-party developers to retrieve the data via an open API. Some servers such as Microsoft HealthVault allow two-way communication, whereas others such as Withings do not allow the uploading of data from a third-party app. Some servers only present the collected data in graphical or table format, whereas others analyze the data and provide trend analysis and various insights.

In this paper, we focus mainly on sensor-generated health and fitness data, but it is worth mentioning that 80% of myFitnessCompanion users enter their physiological data manually [27]. We suspect that most users use their existing blood pressure monitor or weight scale devices that are not wirelessly enabled and transfer the readings manually to the app.

## Results

### Overview

The main result of this paper is a health and fitness app called myFitnessCompanion. The results and discussions in this paper are based on our experience as an integrator of health data from various sources. The app has over 6000 users. Screenshots of the myFitnessCompanion app are shown in Figure 2 and a video showing the app's functionalities is shown in Multimedia Appendix 1.

**Figure 2:**
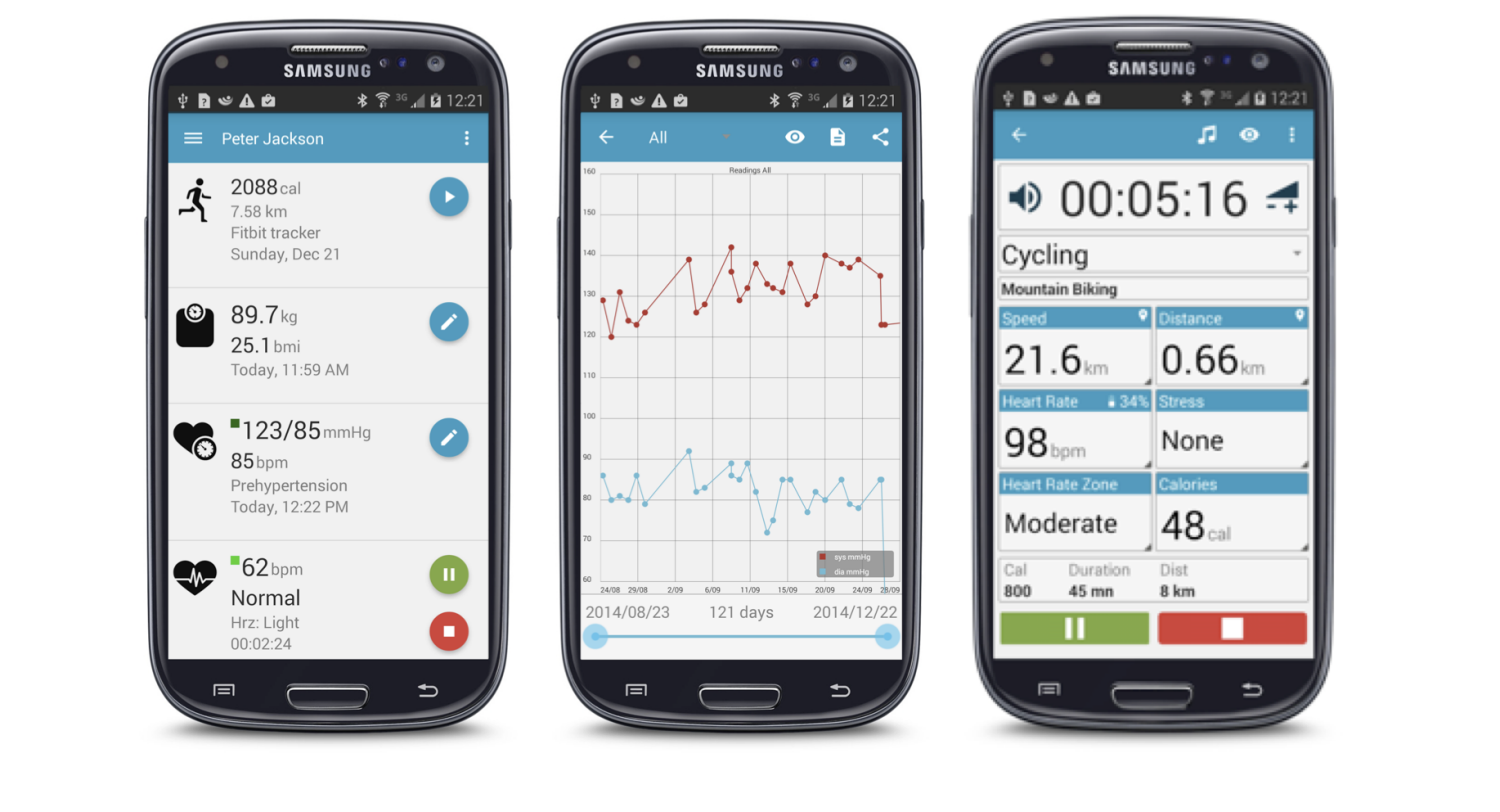
Screenshots from the myFitnessCompanion app.

### Technical Challenges Integrating Wireless Devices

myFitnessCompanion has integrated a wide variety of wireless sensors ranging from universal serial bus (USB) cable devices to the latest Bluetooth low energy (BLE) devices. We focus on the most commonly used wireless communication protocols.

#### Classic Bluetooth

Devices that have been on the market for several years mostly use classic Bluetooth. Most mobile phones support classic Bluetooth, whereas only the later and more expensive models support BLE, the latest version. Classic Bluetooth supports different ways to communicate between a device and a mobile phone. We encountered all possible options, which resulted in writing specific software for each device. For example, the A&D weight scale and blood pressure monitor would only activate Bluetooth after a reading is taken. This means that the mobile device has to listen for Bluetooth requests coming from an A&D device and then establish a Bluetooth link. Other devices act as slaves where the mobile phone (master) has to initiate the Bluetooth communication. Yet other devices would alternate between master mode for configuration purposes and then switch to slave mode when data need to be exchanged with the mobile device. In order to integrate a Bluetooth device, we required the protocol specification from the vendor. Dealing with all these different Bluetooth communication modes made the software development complex. Once the Bluetooth communication was solved, the next challenge was to interpret the data received and the data to be sent to the device.

**Figure 3:**
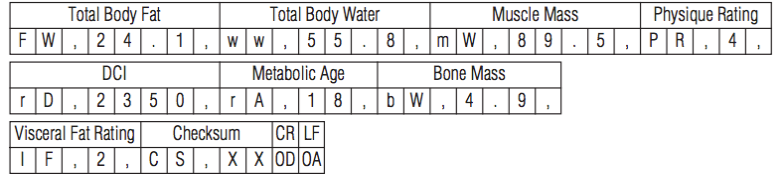
Example of a plain ASCII protocol (Tanita [28] BC590-T scale).

**Figure 4:**
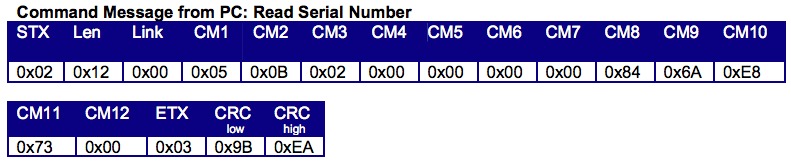
Example of a more complex protocol (OneTouch UltraMini [29]).

Without exception, all vendors developed their own protocol and data formats to retrieve data from the device or to send commands to the device. Some protocols were straightforward, using plain American Standard Code for Information Interchange (ASCII) text to send or receive data. The Tanita [28] BC590-T scale ASCII protocol is seen in Figure 3. Many vendors, however, implemented complex protocols with numerous commands to control and exchange data. Figure 4 shows the more complex protocol for the OneTouch UltraMini [29]. Without a detailed specification, it is impossible to communicate with these devices.

From our experience, devices using classic Bluetooth to stream data continuously (eg, heart rate) are the most reliable from a connectivity point of view. Devices that only activate Bluetooth after a reading have turned out to be unreliable, especially if a mobile device is not in the area. Often the device would not establish a Bluetooth connection on subsequent readings and the user would be forced to go through the pairing process again.

#### Bluetooth Low Energy

The latest health and fitness devices use BLE (aka, Bluetooth Smart or Bluetooth 4.0). Fitbit and Jawbone activity trackers use BLE due to low power consumption while maintaining a similar communication range compared to classic Bluetooth. BLE is characterized by easy pairing with a mobile device and minimal or no user intervention required. Many BLE devices such as heart rate monitors start transmitting automatically when data are available. BLE is rapidly becoming the standard for wearable devices, pushing ANT+ to the background. BLE has built-in features to automatically reconnect to a mobile device if the connection is lost. This eases software development and improves the reliability of device-phone communication. The introduction of BLE, together with standardized protocols for data exchange, makes these devices easy to integrate and use.

However, several vendors such as Fitbit and Withings decided to use proprietary protocols, making it impossible for third-party developers to communicate directly with their devices. We believe that this will change in the near future with other vendors offering similar devices using open protocols. Android Wear and the upcoming Angel [30] wearable device already allow developers to read the data directly from the device. In particular, new releases of Android Wear devices will offer the same (and more) functionality as Fitbit trackers and we believe this will force these vendors to open their devices or lose market share.

#### Adaptive Network Technology+

ANT+ is a lesser-known wireless technology. It is characterized by low power consumption and short-range communication. It is mainly used in sports-related devices, such as step counters, fitness equipment, and heart rate monitors. It is similar to BLE, but not many mobile phone makers integrate ANT+ communication into their phones, therefore limiting the popularity of ANT+ devices. ANT+ devices implement a standardized protocol, which makes it easy to integrate these devices.

We believe that over time the market will converge on BLE at the cost of classic Bluetooth and ANT+. BLE is a natural evolution of classic Bluetooth and already the latest mobile phones support BLE and not ANT+. This will force health and fitness device vendors to support BLE if they want to have a slice of the booming health and fitness device market.

#### Sensor Data Duplication

myFitnessCompanion can support up to seven active sensors at the same time. It is not common, but customers do use multiple sensors simultaneously. For example, sleep apnea patients use a heart rate monitor and a pulse oxygen sensor concurrently, which results in duplicate heart rate readings varying slightly. Currently, our app records both heart rate readings and tags the source of the readings, which gives the user an indication in case of discrepancies. In future versions, our app will give the user the option to select which sensor should be used for real-time analysis and feedback. With the increase of data sources comes the need to be able to differentiate the sources based on their reliability, quality, and trust levels.

#### Sensor Data Reliability

The reliability of the devices varies widely, partially caused by incorrect use by the user. This is a major concern for health professionals when customers present, for example, their blood pressure readings expecting a health professional to make a diagnosis based on self-collected health data. Devices made for the fitness market are not necessarily approved by the Food and Drug Administration (FDA) and, as such, are even less reliable. Currently, myFitnessCompanion cannot identify the quality of a sensor reading; however, it tags the source of the reading. Knowing the source of the data collected is beneficial for a health professional in his/her assessment of the data quality.

### Technical Challenges Integrating Back-End Servers and Electronic Health Records

myFitnessCompanion can upload and download health data from various servers, such as Microsoft HealthVault, Google Fit, Jawbone, Fitbit, and many more. These servers offer an open API where (after authorization) health data can be exchanged. In this discussion, we focus on authorization and use of standards for the exchange of health data.

#### Authorization

**Figure 5:**
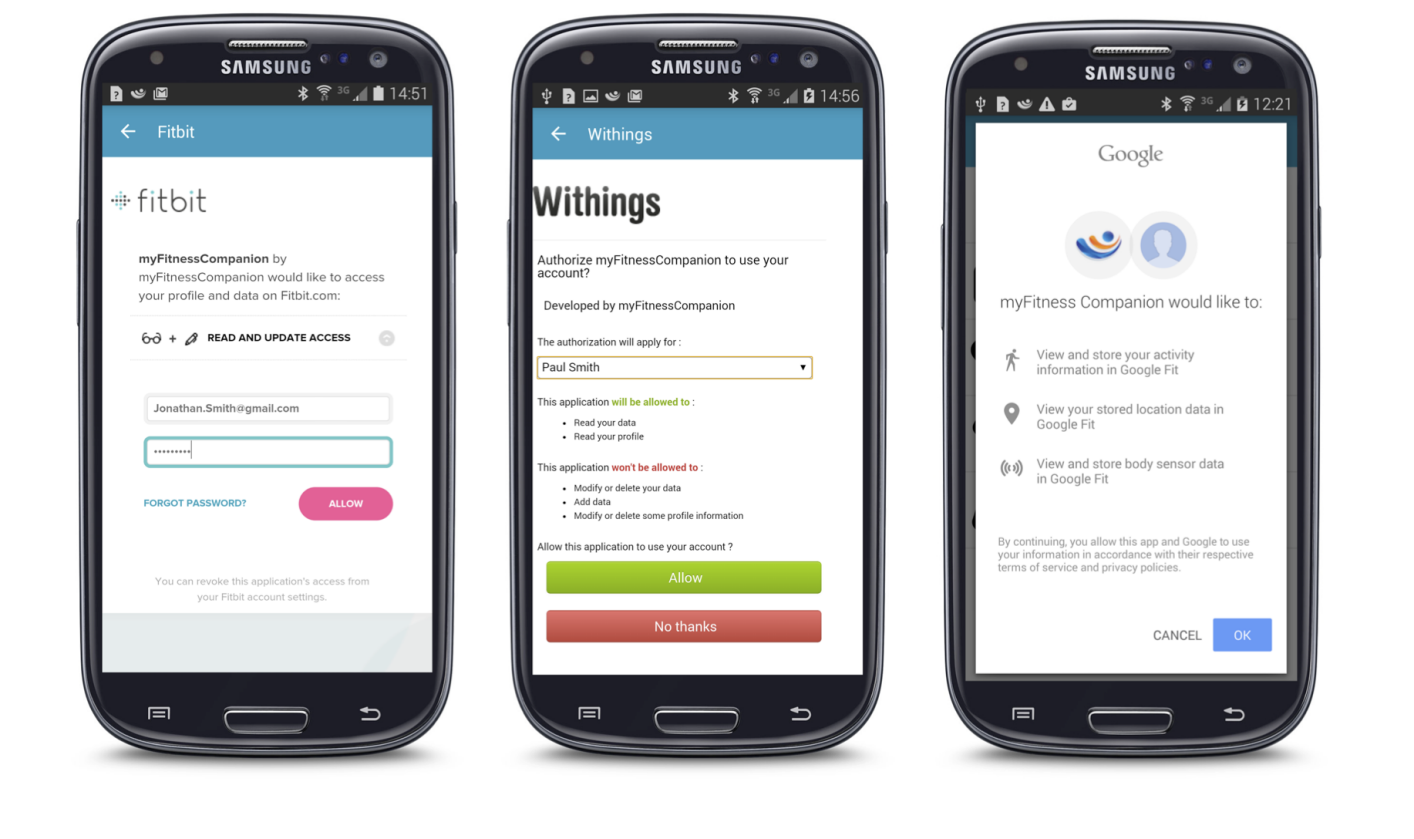
Screenshots of the OAuth for Fitbit (left), Withings (middle), and Google Fit (right).

All servers use some version of open authorization (OAuth). OAuth is an open standard and provides apps like myFitnessCompanion secure delegated access to a server on behalf of the owner. OAuth specifies a process to authorize third-party access to the resources without sharing the user credentials. Once a user has given myFitnessCompanion permission to access health data on their behalf, the app can download and upload data without further user intervention. Figure 5 shows screenshots of the OAuth for Fitbit, Withings, and Google Fit.

Although OAuth is a well-defined standard, the actual implementation varied slightly for the different servers. For example, the FatSecret server supports the OAuth 1.0 specification, but in their actual implementation they used variable names that differ from the standard. The consequence was that off-the-shelf libraries for OAuth for Android devices could not be used and custom software had to be written to deal with these slight discrepancies.

Microsoft HealthVault uses yet another variant of OAuth and specific libraries needed to be used in order to be able to communicate with the HealthVault server. In addition, some servers implement the OAuth 1.0 version whereas others support OAuth 2.0. All this added up to additional complexity of the software to deal with the various servers. A positive trend is that servers are migrating toward OAuth 2.0, so we can expect in the near future to use one standard for authorization.

#### Health Level Seven Compliance

Once the authorization hurdle had been overcome, the next challenge was to deal with the actual data to be exchanged between myFitnessCompanion and a server. Unfortunately, not a single server used an official standard for health data exchange. Without exception, each server defined its own specific data format. All the efforts made by the Health Level Seven (HL7) standardization group seem to be ignored and not taken into account. The API offered by Microsoft HealthVault is the closest to something that looks like an HL7 specification, but a specific subset has been used with proprietary modifications. The consequence was that each server-specific software had to be written to interpret the data.

#### JavaScript Object Notation Versus Extensible Markup Language

**Figure 6:**
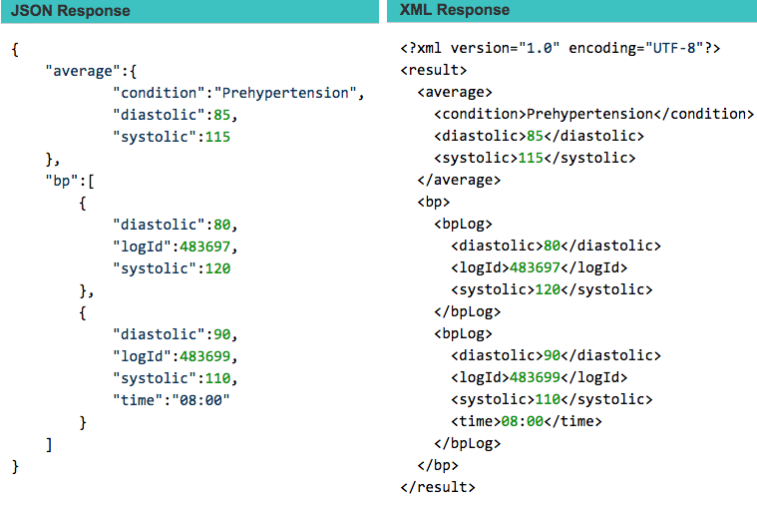
Example responses using JSON (left) and XML (right).

On a positive note, most servers offer their data in either Extensible Markup Language (XML) or JavaScript Object Notation (JSON) format, with JSON rapidly becoming the de facto standard. We expect that XML will disappear in the next few years. Fitbit has stopped offering the XML API in 2015 and will only support JSON. Only Microsoft HealthVault solely uses XML and does not offer JSON, which makes it much harder for developers to convert the data into a usable format for further processing. Figure 6 shows example responses using JSON and XML.

#### Server Data Duplication

myFitnessCompanion supports a two-way synchronization where data can be uploaded to, and downloaded from, a server. Dealing with one server is fairly straightforward, but issues arise when data need to be synchronized using multiple servers. Should data that originated from, for example, the Fitbit server be duplicated to HealthVault and Jawbone servers, or should the data only be imported to the mobile app and not uploaded to other servers? Because of the API specification of some servers, it is impossible to identify where the data originally came from, so if you upload it to another server it becomes a new reading and imported again into myFitnessCompanion, resulting in duplicates. To avoid this issue, when myFitnessCompanion imports readings from a server, it does not upload these readings to other servers. This means that the app becomes the central point where data from various sources come together.

## Discussion

### Principal Findings

The result of this research is a health and fitness app, myFitnessCompanion, which is able to aggregate data from multiple sources—activity trackers, wireless sensors, and servers—and analyze and present the data in a personalized manner. Over 6000 users use the app worldwide to aggregate their health and fitness data. It demonstrates that mobile apps can be used to enable interoperability. Challenges encountered in the research process included the different wireless protocols and standards used to communicate with wireless devices, the diversity of security and authorization protocols used to be able to exchange data with servers, and lack of standards usage, such as HL7 for medical information exchange.

In terms of interoperability, we have achieved three levels of interoperability: foundational (the app and EHR can exchange data), structural (the data can be interpreted at the field level of exchange), and semantic (the data can be exchanged and used by both the app and the EHR). If we refer to the six levels of the refined eHealth European Interoperability Framework (eEIF) model [31], we address the technical (ie, apps and IT infrastructure) and semantic aspects. We cover organizational (ie, policy and care process) and legal interoperability aspects for the private clinical EHR systems we interoperate with. For these systems, there are privacy and security measures in place to obtain user trust and acceptance of the complete ecosystem [32].

### Limitations

myFitnessCompanion has been developed for the Android platform. An Apple iOS and Windows Mobile version would be desirable to cover the majority of mobile devices. Currently, the aggregated data reside on a mobile device or are sent to private EHR systems. It would be desirable to have these data stored in government-controlled EHR systems. Unfortunately, tapping into official EHR systems turned out to be complicated. Efforts have been made to connect myFitnessCompanion to Australia's personally controlled EHR (PCEHR) system, but they failed. The PCEHR standards are too complex and difficult to implement. There is no support and no easy-to-use API to interact with the PCEHR. The new version, called My Health Record, may deal with this issue. Other official EHR systems have security and operational policies that are not coherent with other systems and they do not allow any third-party developer to tap into the system [33]. This makes the integration of the two health data streams complicated and bridging the gap requires time and cooperation from governments to allow third-party developers to tap into their systems on behalf of its users.

Acceptance by health professionals is another hurdle to overcome. From user feedback, we know that users show their collected health data (eg, blood pressure and blood glucose readings) to their health professionals. Some health professionals take this data into account for the diagnosis, but others reject the self-collected data and use their own (often far more limited set of data) for diagnosis. Reasons for rejection include the potential lack of accuracy and the extra time needed to go through the data [34]. There is a need to apply some data mining or filtering techniques to extract the important information from the vast amount of data and save precious time. Once this is in place, we believe that more health professionals will accept self-collected health data, especially if the source of the biometric data is properly tagged and they know where the data came from. It is important that the data are fit for the purpose (eg, fitness trackers to identify the level of activity). A study involving 1406 health care providers in the United States [35] highlights that their acceptance depends on the type of data collected. For example, 60.60% of these health care providers would trust a mobile phone for heart rate information.

Another survey of 1000 American health professionals [36] found that 42% of physicians were comfortable relying on at-home test results to prescribe medication and nearly 66% of physicians would prescribe an app to help patients manage chronic diseases such as diabetes. In addition, 86% of clinicians believe mobile apps will become important for them to manage their patients’ health over the next 5 years.

### Comparison With Prior Work

There are a lot of health and fitness apps on the market, and some good state-of-the-art analyses of those apps can be found in various studies [37-39]. An excellent review on the requirements for, and barriers toward, interoperable eHealth technology in primary care can be found in Nijeweme-d'Hollosy et al [40]. Only a few apps address interoperability and are real aggregators of health and fitness data; a research report [41] has identified those apps as the connected mHealth app elite, and positioned myFitnessCompanion in the top five of this group. Google Fit claims to be an aggregator of health data, but its current version is limited to fitness data only. Apple’s HealthKit is more promising, storing a wide variety of health and fitness data, but is limited to an Apple ecosystem.

### Conclusions

As stated in the “Introduction” section, a combination of informal health and fitness data and official health data stored in EHR systems is desirable to provide a complete health picture. myFitnessCompanion is able to tap into both the formal and the informal health and fitness data, and aggregate the data in one place. There are a lot of benefits in aggregating the data coming from wearable devices and sensors, especially, for example, for users with chronic disease, as their conditions need long-term monitoring. By combining health data with nonhealth data (eg, location, social media, and habits), one can make interesting correlations and suggest changes to the users’ habits and help in dealing with their chronic conditions. Our ultimate objective is to empower users and help them in monitoring their health and fitness in a personalized manner and to improve their quality of life [42]. myFitnessCompanion has the potential to change health care by empowering users and helping them take control of their health.

## Appendix

Multimedia Appendix 1 Video showing the functionalities of myFitnessCompanion.

## Abbreviations

ANT: Advanced and Adaptive Network Technology
API: application programming interface
ASCII: American Standard Code for Information Interchange
BLE: Bluetooth Low Energy
eEIF: eHealth European Interoperability Framework
EHR: electronic health record
FDA: Food and Drug Administration
HL7: Health Level Seven
IMS: Intercontinental Marketing Services
iOS: iPhone operating system
JSON: JavaScript Object Notation
OAuth: open authorization
OS: operating system
PCEHR: personally controlled EHR
USB: Universal Serial Bus
XML: Extensible Markup Language
